# The Effects of Acute Bisphenol A Toxicity on the Hematological Parameters, Hematopoiesis, and Kidney Histology of Zebrafish (*Danio rerio*)

**DOI:** 10.3390/ani13233685

**Published:** 2023-11-28

**Authors:** Svetlana Smorodinskaya, Nikita Kochetkov, Kirill Gavrilin, Dmitry Nikiforov-Nikishin, Diana Reznikova, Aleksey Vatlin, Anastasia Klimuk, Maya Odorskaya, Alexei Nikiforov-Nikishin, Andrey Ponomarev, Maria Marsova, Valery Danilenko

**Affiliations:** 1 Laboratory of Bacterial Genetics, Vavilov Institute of General Genetics Russian Academy of Sciences, 119333 Moscow, Russia; kler.smo@gmail.com (S.S.); niknikdl@rambler.ru (D.N.-N.); reznikova.da@phystech.edu (D.R.); vatlin_alexey123@mail.ru (A.V.); klimukanastasia27@gmail.com (A.K.); maya_epifanova@mail.ru (M.O.); valerid@vigg.ru (V.D.); 2 Faculty of Biotechnology and Fisheries, Moscow State University of Technologies and Management (FCU), 73, Zemlyanoy Val Str., 109004 Moscow, Russia; k.gavrilin@yandex.ru (K.G.); 9150699@mail.ru (A.N.-N.); ponomarev777@inbox.ru (A.P.); 3 Phystech School of Biological and Medical Physics, Moscow Institute of Physics and Technology, Institutsky Lane 9, 141700 Dolgoprudny, Russia; 4 Institute of Ecology, Peoples’ Friendship University of Russia (RUDN University), 117198 Moscow, Russia

**Keywords:** LC50, hematopoiesis, head kidney, histology, genotoxicity, Bisphenol A, *Danio rerio*

## Abstract

**Simple Summary:**

Bisphenol A (BPA) is an organic compound used in the manufacture of plastic products and is considered a common pollutant in the aquatic environment. Its impact on aquatic organisms is manifested in several reactions, particularly in changes in peripheral blood and disorders of hematopoiesis. In the present study, the effects of high concentrations of BPA on several indices of zebrafish blood were investigated. Differences in the effects of high and low concentrations on blood composition and disorders in hematopoietic organs were identified. The genotoxic effect of BPA at concentrations of 6 and 8 mg/L was confirmed. The test parameters most affected by the toxicant, such as the number of young erythrocytes in peripheral blood, nuclear anomalies of erythrocytes, and disturbances in hematopoiesis processes in the head kidney, expressed as changes in the ratio of immature/undifferentiated cell elements, were determined.

**Abstract:**

In this study, the results of evaluating the acute toxicity of Bisphenol A on *Danio rerio* are presented, encompassing peripheral blood parameters, the composition of hematopoietic cells of erythroid and myeloid lines in the head kidney, and data from histological studies. The LC50 values of Bisphenol A for adult zebrafish individuals for 12, 24, and 48–96 h were determined, which were 18.04, 7.55, and 6.22 mg/L, respectively. The study includes data on the morphology and quantitative frequency of specific cells in the hematopoietic tissue of the head kidney, along with the consideration of adaptive mechanisms in hematopoiesis under BPA exposure. The application of polynomial regression analysis to reveal the concentration–effect relationship for some hematological and histological parameters was demonstrated. Significant increases in the frequency of erythrocyte nuclear abnormalities were observed at BPA concentrations of 6 and 8 mg/L, which indicates a genotoxic effect. BPA’s impact on fish peripheral blood parameters manifested as an increase in the number of erythrocytes (RBC) and immature erythrocytes, as well as a decrease in the number of lymphocytes. The most notable pathological changes in the head kidney’s hematopoietic tissue included circulatory disturbances and the formation of inflammation/degradation foci, as confirmed by histopathologic indices. At BPA concentrations of 2 and 4 mg/L, the observed changes were compensated for by hematopoietic adaptation mechanisms; however, at concentrations of 6 and 8 mg/L, acute systemic toxicity was evident.

## 1. Introduction

Bisphenol A (BPA) is an important component used in the production of technological plastic products, such as pipes, epoxies, and coatings, and in various food packaging [1,2]. The global production level of BPA has been steadily increasing, rising between 1% and 5% per year, indicating a steady demand [3]. The particular interest in BPA is due to its potential harmful effects on humans [4] as well as on aquatic ecosystems. It can enter water bodies through wastewater treatment plant effluents, industrial effluents from petrochemical plants, and landfill leachates [5]. Some species of aquatic organisms, living near plant effluents or river and marine sediments, may be exposed to high concentrations of BPA on a continuous basis [6], thus making it necessary to conduct in-depth studies on its effects.

Numerous studies have that BPA has a negative impact on the environment and aquatic organisms. The toxic properties of BPA are well enough studied, both on invertebrates and fish [6]. Many researchers have noted the genotoxic properties of this compound [7,8], as well as its negative effects on endocrine functions [9], the nervous system, and the digestive and excretory organs in fish [10]. The most significant effect that BPA has may be on the reproductive performance of organisms due to its estrogenic activity [11]. BPA is able to remain active in the aquatic environment for extended periods of time, accumulating in water bodies and aquatic organisms [12]. As a consequence, its acute and chronic toxicity to aquatic species can vary considerably, as acute toxicity is most often manifested through short-term exposure to the substance and is expressed in serious disorders that can lead to death of the organism. During the determination of the acute toxic effect of a pollutant on organ and tissue systems, it is advisable to conduct the study using high concentrations (i.e., lethal and sublethal).

Hematological and biochemical indicators are often used as prognostic and diagnostic methods to assess fish health because of their versatility and high sensitivity [13]. It is worth noting that alterations in the morphological pattern of blood can manifest quite rapidly after the ingestion of a toxicant by the fish organism. Many toxicants induce toxic anemia in fish, the causes of which can be both the disruption of the gill apparatus and changes in the processes of erythropoiesis and deposition of cellular elements [14]. If the toxic effects do not lead to mortality, the blood composition of the fish undergoes significant changes as a result of the activation of the organism’s compensatory reactions. Adaptive responses in the qualitative and quantitative composition of peripheral blood have been studied for many fish species [15,16]. However, there are some discrepancies in the methodology used to account for these changes [17,18].

Hematopoiesis is a complex process in which hematopoietic stem cells, which are the most immature elements of the hematopoietic lineage, proliferate and differentiate into different groups of hematopoietic progenitor cells [19]. The action of the toxicant on the differentiation processes in fish blood cells will be expressed in the disruption of cell kinetics (in the ratio of cells in the hematopoietic tissue, presence in the bloodstream, and deposition locations). For this reason, quantitative indicators of hematopoiesis can be used as a marker of toxic effects.

Laboratory fish species can be used to determine the mechanisms of action of toxicants, as well as target organs. *Danio rerio* is a common test subject for studying the toxicity of various compounds [20] and is also actively used by researchers in studying the processes of hemocytopoiesis [21]. The most active hematopoietic processes in zebrafish occur in the head section of the kidney, although immature cells of the hematopoietic series can be found in the spleen and some other organs (i.e., mucosa-associated lymphoid tissues) [22]. During the phases of cell division and differentiation, cells can be exposed to cytotoxic effects; the visible manifestations of which are the formation of various nuclear abnormalities found in mature cells [23]. The micronucleus test, performed on the peripheral blood erythrocytes of fish, assesses the frequency of occurrence of such nuclear abnormalities in red blood cells and allows the estimation of the genotoxic effect of the examined substance [24]. The micronucleus test is widely used to assess clastogenic and aneugenic effects both in vitro and in ecotoxicological studies [25].

Thus, the aim of this study was to determine the effects of different lethal and sublethal concentrations of Bisphenol A on fish survival in an acute experiment, hematological parameters, and hematopoiesis processes, as well as histological changes in the head kidney of *Danio rerio*.

## 2. Materials and Methods

### 2.1. Animal Maintenance

*Danio rerio* (wild type), 4–5 months old, were kept in 300 L aquariums with 200 fish in each aquarium, equipped with mechanical and biological filtration systems, with 10% water replacement per day. Fish maintenance and care were carried out according to the recommendations of Westerfield [26]. Prior to the experiment, fish were fed with Tetra Min Flakes XL (Spectrum Brands, Melle, Germany).

For the study, individuals of both sexes without visible injuries were selected, with an average size of 2.12 ± 0.3 cm, and a weight of 0.29 ± 0.03 g. During the acute experiment, fish feeding was not performed.

### 2.2. Acute Toxicity Bioassay

To determine lethal concentrations of Bisphenol A (BPA), LC50 (Lethal concentration 50) values for 12–96 h were used. The studies were performed according to OECD Test no. 203 with some modifications. Bisphenol A (2,2-bis(4-hydroxyphenyl)propane) with 99% purity was purchased from Sigma-Aldrich (St. Louis, MO, USA). The concentration range for the acute experiment was selected based on data from the literature on other fish species and included: 0.5, 1.0, 2.0, 4.0, 6.0, 8.0, 12.0, 16.0, and 20.0 mg/L [27,28]. Experiments were carried out in triplicate in 50 L aquaria with 20 zebrafish individuals placed each (*n* = 20 × 3). Temperature (24 °C), light regime (12:12 h), and hydrochemical parameters (pH 7.3 ± 0.3; O_2_ 7.9 ± 0.2; NH_4_ < 0.05; NO_2_ 0.15 ± 0.02; NO_3_ 3.1 ± 0.9) in the experimental tanks at the beginning of the acute experiment were the same as the maintenance aquariums. Changes in behavior, clinical signs, and mortality were monitored throughout the experiment.

Since BPA is poorly soluble in water, dimethyl sulfoxide (DMSO, Tula Pharmaceutical Factory LLC, Tula, Russia) was used as the standard solvent (OECD Test no. 203, [29]) at a concentration of 1% (*v*/*v*) to prepare a stock solution, following the approach of other authors [30]. In the control group (CTR), fish were exposed to DMSO alone.

### 2.3. Preparation of Peripheral Blood and Kidney Smears

After the end of the acute experiments, four zebrafish individuals (*n* = 4) were sampled from the control and experimental groups with Bisphenol A concentrations (2 mg/L or 0.32 LC50; 4 mg/L or 0.64 LC50; 6 mg/L or 0.96 LC50; and 8 mg/L or 1.28 LC50) to prepare blood smears and the hematopoietic tissue of the head kidney. Before sampling biological material, fish were anesthetized in an MS-222 solution (20 mg/L, LGC Mikromol, Luckenwalde, Germany) for 3 min. Blood was collected for the preparation of peripheral blood smears from the caudal vein according to the technique previously described in [31]. Immediately after blood collection, the number of erythrocytes and leukocytes was counted using a hemocytometer [32].

After preparing the blood smears, the dissection of experimental fish was performed following the methodology outlined by Ivanovski et al. [33]. Therefore, upon opening the abdominal cavity of the zebrafish, the organs of the gastrointestinal tract were removed to provide access to the head section of the mesonephros. Kidney tissue samples were extracted using a flat-tipped curette, after which the tissue was spread on a slide and allowed to dry for 12 h.

The prepared smears were stained according to the standard technique [34]. Briefly, after drying for 4 min, smears were fixed in Nikiforov’s mixture (*v*/*v*, methyl alcohol/diethyl ether) and exposed for 2 min, followed by eosin staining with methylene blue for 4 min (Mein-Grunwald Giemsa Staining). Then, the preparations were washed and dried, followed by additional staining with Romanovsky–Giemsa azure–eosin for 10 min. The obtained smears were washed again in containers with distilled water and dried for 30 min.

Smears of blood and the head kidney were viewed under an Olympus BX53 light microscope (Olympus Corporation, Tokyo, Japan) with Carl Zeiss ERc 5s (Zeiss, Oberkochen, Germany) and ToupCam 16.0 MP (ToupTek Photonics, Hangzhou, China) ocular attachments using ZEN lite (Zeiss, Germany) and ToupCam view 16.0 (ToupTek Photonics, China) software.

### 2.4. Assessment of Hemopoiesis Activity and Composition of Peripheral Blood

The following cellular elements were counted in peripheral blood smears: mature and young (juvenile) forms of erythrocytes, lymphocytes, granulocytes, monocytes, and thrombocytes. At least 40 fields of view were examined for each blood sample. The total number of cells counted for one preparation was at least 2500. Peripheral blood cellular elements were counted and determined according to generally accepted methods [13].

Erythropoietic (basophilic erythroblast, polychromatic erythroblast, orthochromatic erythroblast, and erythrocyte) and myelopoietic (promyelocyte, myelocyte, metamyelocyte, bacillary granulocyte, segmented granulocyte) cellular elements, as well as blast cell forms (i.e., erythroblasts, myeloblasts/granuloblasts), were counted on the obtained head kidney smears according to the characteristics described by Ivanova [35], Fijan [36], and Kondera [37]. Cells with features of several undefinable elements were referred to as unclassified cells. Basophilic, eosinophilic, and neutrophilic forms were counted together when counting differentiated forms of granulocytes; the distinction was made based on the morphology of the nucleus (band and segmented form) in accordance with the work of Wlasow and Dabrowska [38]. At least 20 fields of view were examined per smear, and at least 500 cells were counted. Additionally, morphometric parameters (cell, nuclei length/width; Table S1) were assessed using the ImageJ program (Wayne Rasband, NIH; [39]) to determine the different morphological stages of hematopoietic stem cells and hematopoietic progenitor cells.

### 2.5. Micronucleus Test

Erythrocytes with pronounced nuclear abnormalities were counted in prepared fish peripheral blood smears according to the methods [24,40]. In addition to micronuclei (MN), the following nuclear abnormalities were considered: notched nuclei (NN), lobed nuclei (LN), and blebbed nuclei (BN). The detection of several types of nuclear abnormalities allows an increase in the reliability of the genotoxicity assessment [31]. To accurately identify micronuclei and other nuclear anomalies, a number of distinguishing characteristics were used: identical morphology of the nucleus and micronuclei, a small distance from the nucleus, size from 1/16 to 1/3 of the nucleus, and minimal difference in color from the nucleus [34].

### 2.6. Histological Preparations, Histomorphometric Analysis and Semi-Quantitative Assessment

To obtain histologic sections, 4 individuals were selected from each experimental group. Three sections were made from each selected fish (*n* = 4 × 3). The fish were anesthetized in MS-222 solution (10 mg/L) and then fixed in Davidson’s solution for 24 h. Tissue samples were subsequently dehydrated in a series of graded alcohols and embedded in paraffin. Next, total serial sections (4 μm) were prepared in the frontal plane and stained with hematoxylin and eosin (H&E) and periodic acid-Schiff (PAS). The histological sections were prepared and stained following the method described by Suvarna [41].

Histologic sections were examined using the equipment described in Section 2.3. The ImageJ program was used to measure morphometric parameters on histological sections of the kidney. The measurements were performed according to the method described in the previous paper [42].

The histopathologic index (HI) was calculated according to Bernet [43]. Briefly, for each of the 4 types of reaction patterns (circulatory changes, regressive and progressive changes, inflammatory response), a number of histopathologic abnormalities were defined, each corresponding to its own importance factor (ranging from 1 to 3) reflecting the effect of histopathologic changes on organ functions. The value of each histopathologic abnormality was set between 0 and 5, where 0 indicates the absence of changes in the entire slide area and 5 represents the presence of changes in 80–100% of the slide [44]. The index of each reaction pattern was then calculated by multiplying the histopathologic abnormality score by its importance factor and summing them for each reaction pattern.

### 2.7. Statistical Analysis

Comparison data of the analyzed variables are presented as mean ± SD. Statistical significance was determined using nonparametric tests (Kruskal–Wallis test, Mann–Whitney U-test), based on the distribution of the data and the homogeneity of variances (assessed with the Shapiro–Wilk and Levene tests). A *p*-value < 0.05 was considered statistically significant. The correlation between the studied parameters and toxicant concentration was determined using Pearson correlation. In the case of obtaining a heterogeneous structure of parameter dependence on toxicant concentration and a low regression coefficient, polynomial regression was used.

The median lethal concentration was calculated graphically using probit analysis [45]. The 95% confidence intervals (CI) for the obtained LC50 values were also determined.

Statistical analyses were conducted using GraphPad Prism version 9.0 software (GraphPad, San Diego, CA, USA) and R software (v3.5.2)/RStudio [46,47].

## 3. Results

### 3.1. Determination of LC50

We conducted the acute experiment in a wide range of concentrations of Bisphenol A, allowing us to establish the calculated LC50 values (Table 1). At the maximum studied concentrations (16 and 20 mg/L), mortality began to occur at 12 h of the experiment. The acute toxic properties of the experimental toxicant were manifested on the first day at concentrations above 8 mg/L. At concentrations of 6 and 8 mg/L, fish death was observed at 48 h, while no further deaths were found during the remaining period of the experiment.

**Figure 1:**
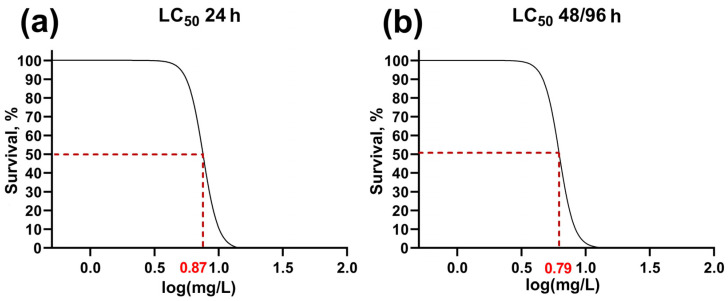
Determination of LC50 values of Bisphenol A for *Danio rerio* adults for 24 (**a**) and 48/96 h (**b**).

BPA concentrations below 6 mg/L did not result in significant fish deaths for the entire duration of the experiment. The obtained LC50 values at 24 and 48/96 h are shown in Figure 1, and the numerical data and conversion to mg/L are summarized in Table 1. The LC50 value at 48 and 96 h was 6.22 mg/L (CI 6.11–6.33).

### 3.2. Hematopoiesis in the Head Kidney

**Figure 2:**
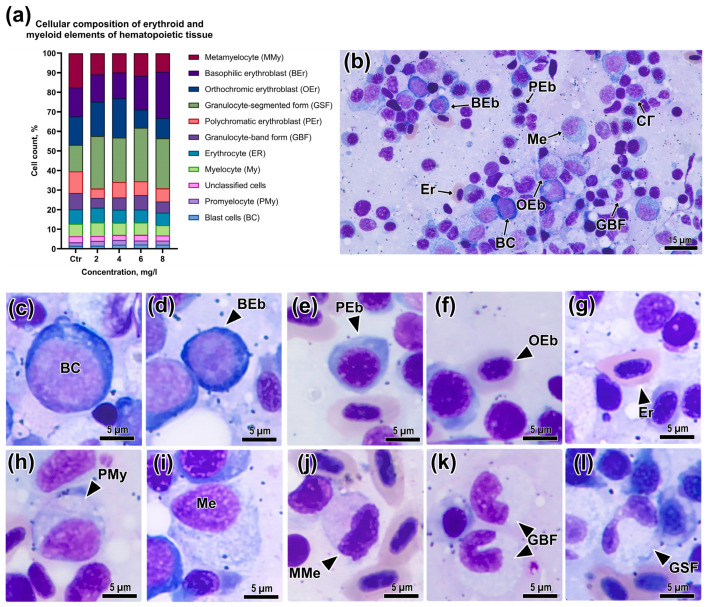
(**a**) Ratio of cellular elements of erythroid and myeloid lineages of hematopoietic tissue of *Danio rerio* head kidney: (**b**) microphotographs of individual stages of hematopoietic cells (**b**–**l**). BC—blast cell, BEb—basophilic erythroblast; PEb—polychromatic erythroblast; OEb—orthochromatic erythroblast; Er—erythrocyte; PMe—promyelocyte; Me—myelocyte; MMe—metamyelocyte; GBF—band form granulocyte; GSF—segmented form granulocyte. May–Grunwald–Giemsa staining. Scale bar: 15 μm (**b**) and 5 μm (**c**–**l**).

The effect of Bisphenol A exposure on the processes of hematopoiesis was studied after a 96 h exposure at concentrations of 2, 4, 6, and 8 mg/L. The general appearance of *D. rerio* head kidney smears and the cells present at different stages of cytodifferentiation are shown in Figure 2b. The occurrence of erythroid elements in the control group was 50.2%, relative to the other cells studied. Basophilic and orthochromatic erythroblasts (14.70 and 14.58%) had the highest occurrence among the blast forms of erythrocytes. The occurrence of erythrocytes in the head kidney smears of the control fish was 7.34%. The total number of myeloid elements was 44.47%. Metamyelocytes were most frequently detected in the smears, the occurrence of which was 14.4%. The list of abbreviations used for hematopoietic cells, hematological, and histological parameters is given in Table S6.

Blast cells (erythroblasts, granulo/myeloblasts) had a large nucleus occupying most of the cell, which could be shifted to the edge, and the cytoplasm was brightly basophilic staining (Figure 2c). Some cells had an oval/kidney-shaped nucleus and small vacuoles in the cytoplasm.

Morphological changes in the erythroid cell during development included: a decrease in the degree of basophilia of the cytoplasm; a decrease in the size of the cell and nucleus (as a result of chromatin condensation) and the nucleus/cytoplasm area ratio; a change in the cell shape toward ellipsoidal. The basophilic erythroblast had a smaller size than the blast cells (Table S1), a larger cytoplasm volume, and also showed bright basophilic staining (Figure 2d). The cell nucleus was round shaped, with granular chromatin. The polychromatic erythroblast had a lighter cytoplasm (light blue/gray), with a rounded or oval nucleus and individual clumps of chromatin (Figure 2e). This erythrocyte precursor also showed a decrease in cell area compared to the previous stages. The last erythrocyte precursors distinguishable on kidney smears, the orthochromatic erythroblast, had an oval nucleus with compacted chromatin, and the cytoplasm was pink or gray in color (Figure 2f). Compared to the mature erythrocyte, the nucleus was less condensed.

The promyelocytes had slightly basophilic granular cytoplasm, and an oval-shaped heterochromatin nucleus shifted to the periphery, occupying about half of the cell (Figure 2h). The myelocytes had a larger cytoplasm size and, consequently, a larger nucleus/cytoplasm area ratio (Figure 2i). The oval or deformed nucleus also had predominantly heterochromatin content with small clumps of euchromatin. Metamyelocytes were characterized by a more elongated nucleus that could occupy up to half of the cell (Figure 2j). The cytoplasm had either basophilic staining with granular inclusions or weakly acidophilic staining with less granulation. Mature granulocytes/myelocytes had a deformed nucleus of two types: band form (Figure 2k) and segmented form (Figure 2l). The ratio of cytoplasm and nucleus area, as well as cytoplasm staining, could vary depending on the cell type.

At high concentrations of the toxicant (6 and 8 mg/L), significant changes in the ratio of erythropoietic and myelopoietic cells were observed (Table 2; Figure S1). The maximum concentration of the toxicant led to a substantial increase in the number of basophilic erythroblasts (*p* < 0.05). At the same time, there was a significant decrease in the occurrence of orthochromatic erythroblasts relative to the concentrations of 2 and 4 mg/L (*p* < 0.05). All studied BPA concentrations above 4 mg/L resulted in a considerable increase in the occurrence of polychromatic erythroblasts (*p* < 0.05). The number of mature erythrocytes in the hematopoietic tissue of the head kidney of experimental *D. rerio* individuals did not change significantly in any of the experimental groups.

BPA had a meaningful effect on the occurrence of myeloid cells, leading to a decrease in their number compared to the control. A significant effect was detected on the number of cells such as metamyelocytes and segmented and band form granulocytes (Table 2). Thus, a substantial decrease in the relative occurrence of two forms of granulocytes was observed at a concentration of 2 mg/L (*p* < 0.05).

### 3.3. Peripheral Blood Composition

**Figure 3:**
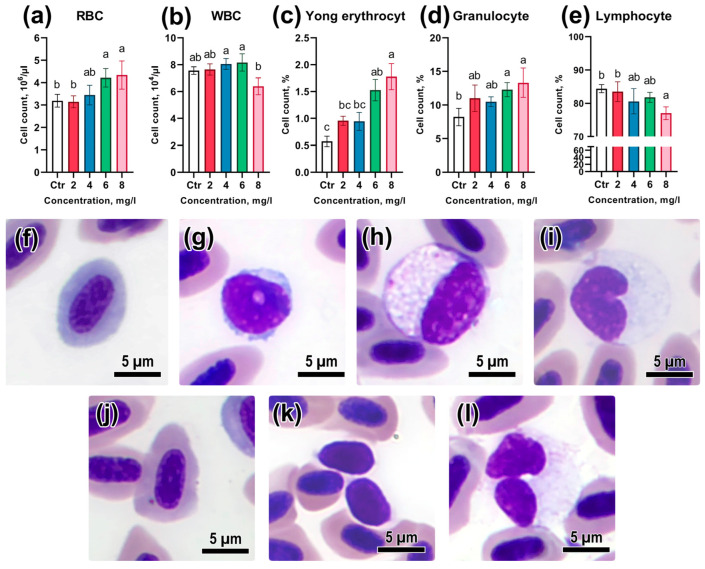
(**a**–**e**) Cell composition of peripheral blood of *Danio rerio* under acute exposure to Bisphenol A: (**f**,**j**)—young erythrocytes at different stages of differentiation; (**g**)—lymphocyte; (**h**,**l**)—granulocytes with different morphology of the nucleus; (**i**)—monocyte; (**k**)—thrombocyte. Significance (*p* < 0.05) from the Kruskal–Wallis test. Superscript letters (a, b) indicate statistical significance between different experimental groups. May–Grunwald–Giemsa staining. Scale bar of 5 μm.

An assessment of the effects of acute toxic exposure to BPA on peripheral blood showed significant changes in the composition and number of erythrocytes and leukocytes (Table S2). The number of red blood cells (RBC) at concentrations of 6 and 8 mg/L considerably exceeded the control values (*p* < 0.05; Figure 3a). Meanwhile, the occurrence of young red blood cells in peripheral blood was also significantly higher than the control values at the respective pollutant concentrations (*p* < 0.05; Figure 3c). Young erythrocytes in peripheral blood, compared to mature ones, had a rounder nucleus, with large chromatin lobules, and weak basophilic staining. Mature erythrocytes had a more pronounced oval-disc shape with an oval nucleus and eosinophilic cytoplasm. It is worth noting that the number of white blood cells (WBC) recorded at the concentration of 8 mg/L was not significantly different from the control; however, it was substantially lower than at concentrations of 4 and 6 mg/L (*p* < 0.05; Figure 3b). The relative number of lymphocytes decreased with increasing BPA concentration, differing meaningfully from the control only at the maximum concentration of the toxicant (*p* < 0.05; Figure 3e). In turn, the occurrence of granulocytes and monocytes had an opposite trend: their number increased significantly with increasing BPA concentration (Figure 3d).

### 3.4. Assessment of Genotoxicity

**Figure 4:**
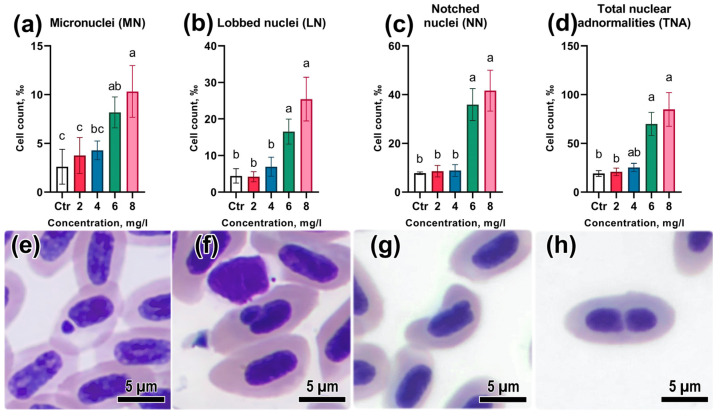
(**a**–**d**) Relative abundance of *Danio rerio* erythrocytes with nuclear abnormalities under acute exposure to Bisphenol A: (**e**) micronuclei; (**f**) blebbed nuclei; (**g**) lobed nuclei; (**h**) notched nuclei. Significance (*p* < 0.05) from the Kruskal–Wallis test. Superscript letters (a–c) indicate statistical significance between different experimental groups. May–Grunwald–Giemsa staining. Scale bar of 5 μm.

Evaluation of the occurrence of nuclear abnormalities in erythrocytes showed that high concentrations of BPA cause a significant increase in the number of erythrocytes with abnormal nucleus morphology (Table S3). The total number of nuclear abnormalities in the control group was at the level of 10.3‰, which is consistent with earlier data on the occurrence of spontaneous nuclear abnormalities in zebrafish [31]. The relative number of micronuclei at concentrations of 6 and 8 mg/L was considerably higher than the control values and values at concentrations of 2 and 4 mg/L (*p* < 0.05; Figure 4e). The occurrence of other nuclear abnormalities in the groups during BPA exposure at concentrations of 6 and 8 mg/L was also significantly higher than in the controls (*p* < 0.05; Figure 4b,c). Total nuclear abnormality (TNA) counts showed that the genotoxic effect of BPA was reliably evident starting at a concentration of 6 mg/L (*p* < 0.05; Figure 4d).

### 3.5. Histologic Parameters of the Kidneys

**Figure 5:**
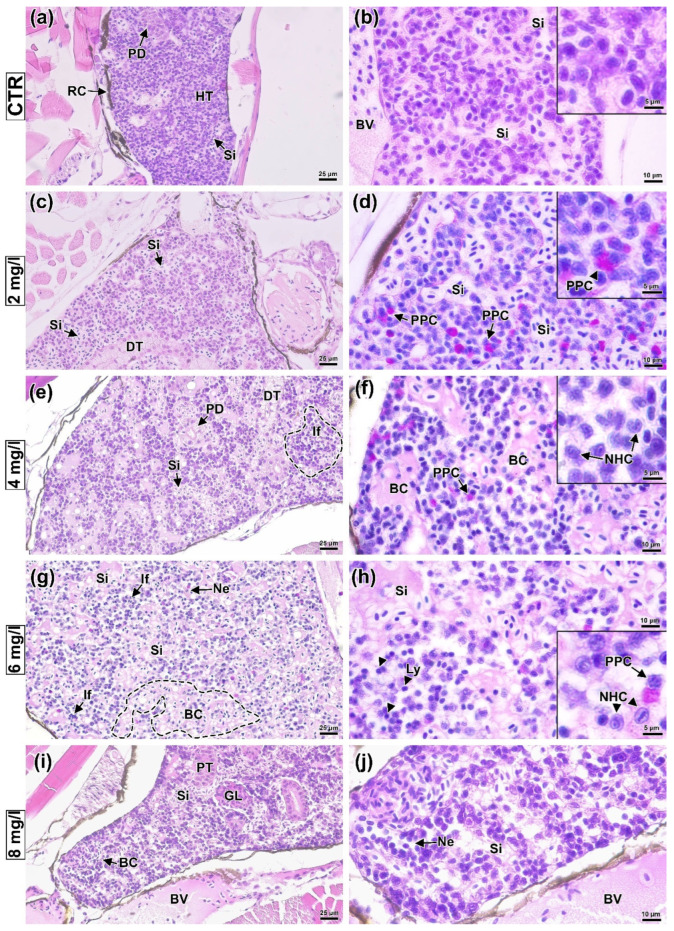
Histologic abnormalities in the hematopoietic tissue of *Danio rerio* head kidney exposed to BPA. CRT (**a**,**b**): normal structure of hematopoietic tissue (HT) surrounded by the renal capsule (RC) and including sinusoidal spaces (Si), blood vessels (BV), and areas of distal (DT), proximal tubules (PT), and glomeruli (GL). In the 2 mg/L group (**c**,**d**): sinusoidal spaces dilated, as well as an increase in the number of PAS-positive cells (PPC) in hematopoietic tissue, were observed. In the 4 mg/L group (**e**,**f**): enlarged sinusoids with evidence of blood congestion (BC), as well as small foci of inflammation (If, dashed line) in hematopoietic tissue, including necrotic cells (NHC). In the 6 mg/L group (**g**,**h**): significant sinusoidal enlargement with blood congestion (dashed line) throughout the head kidney; foci of inflammation and necrosis (Ne) associated with lymphocyte-like cells (Ly). In the 8 mg/L group (**i**,**j**): architectural and structural tissue alterations expressed as impaired circulation (sinusoidal dilation, blood congestion) and necrosis of hematopoietic tissue. H&E (**a**–**c**,**e**,**i**,**j**) and PAS (**d**,**f**–**h**) staining. Scale bar of 25 μm (**a**) and 10 μm (**b**–**f**).

The study of histological sections of *D. rerio* head kidney after exposure to a BPA solution allowed us to establish a number of pathological changes, the significance of which increased with the concentration of the toxicant. Sections of the head kidney of control fish showed hematopoietic tissue consisting of various hematopoietic progenitor cells with sinusoidal spaces between them (Figure 5a,b). In the caudal direction, sections of the nephron, represented by renal tubules and glomeruli, could be seen.

In the group exposed to a BPA concentration of 2 mg/L, there was an increase in the sinusoid width (Figure 5c), as well as an increase in the occurrence of PAS-positive cells (Figure 5d). Increasing the concentration to 4 mg/L revealed an elevated occurrence of circulatory abnormalities, expressed as the enlargement of sinusoidal spaces and blood congestion (Figure 5e). Additionally, foci of inflammation, associated with the disruption of hematopoietic tissue structure and the occurrence of necrotic cells, were observed in some areas (Figure 5f). Necrotic cells from the hematopoietic tissue had chromophoric cytoplasm and disformed karyorrhexis or pyknotic nuclei. A concentration of 6 mg/L led to a further increase in the number of foci of inflammation and necrosis of the hematopoietic tissue. Areas with blood congestion in sinusoids had a diffuse distribution (Figure 5g,h). The occurrence of PAS-positive cells was substantially lower than at concentrations of 2 and 4 mg/L. Significant architectural and structural tissue alterations were expressed in necrosis of hematopoietic tissue and impaired circulation (blood congestion, sinusoidal dilation) were observed at the maximum studied concentration of BPA.

**Figure 6:**
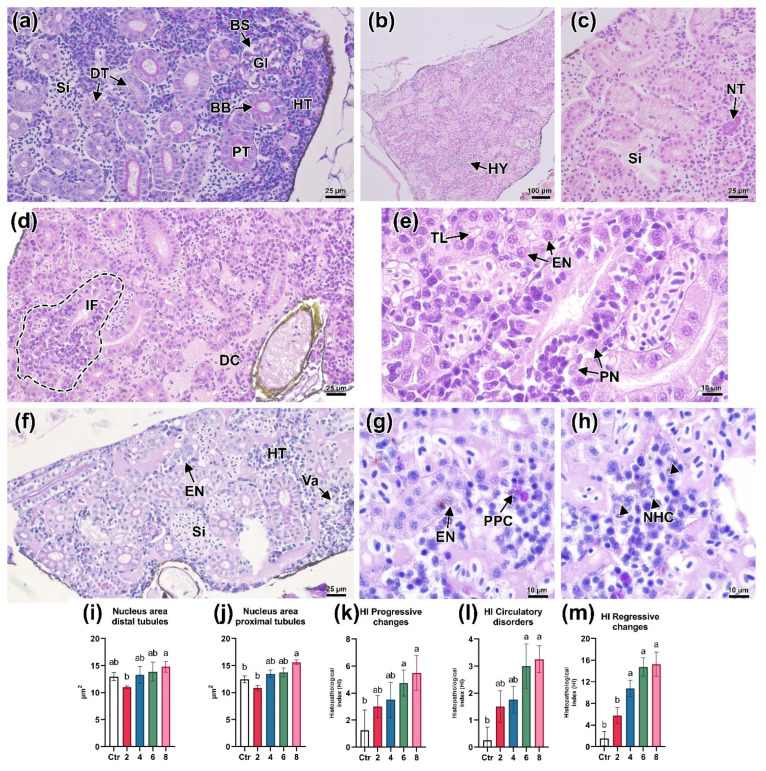
Effect of BPA on nephron structure, hematopoietic tissue, morphometric parameters (**i**,**j**) and histological index (**k**–**m**) of *Danio rerio* trunk kidney. CRT (**a**): normal kidney morphology, distal (DT) and proximal tubules (PT) with a PAS-positive brush border (BB), glomerulus (Gl), and the hematopoietic tissue (HT) and sinusoidal spaces (Si) separating them can be distinguished on the slides. In the 2 mg/L group (**b**,**c**): a significant increase in sinusoid size indicating organ hyperemia (HY); some sections showed an increased occurrence of neonephrogenic tubules (NT). In the 4 mg/L group (**d**,**e**): inflammation foci (If, dashed line) in the area of distal tubule destruction with pycnotic nuclei (PN); marked blood congestion (BC) was seen throughout the slide; individual proximal tubules showed enlarged nuclei (EN) and narrowing of the lumen (TL). In the 6 mg/L group (**f**–**h**): increased nucleus area and vacuolization of renal tubule epithelium (VA); structural abnormalities of hematopoietic tissue as a result of circulation disruption (sinusoidal dilation) as well as necrotic hematopoietic cells (NHC, arrowhead); individual PAS-positive cells (PPC) were found in areas with degradation of hematopoietic tissue. H&E (**b**–**e**) and PAS (**a**,**f**–**h**) staining. Scale bars are 100 μm (**b**), 25 μm (**a**,**c**,**d**,**f**), and 10 μm (**e**,**g**,**h**). Significance (*p* < 0.05) from the Kruskal–Wallis test. Superscript letters (a, b) indicate statistical significance between different experimental groups.

Studying the kidney tissue of *D. rerio* allowed us to reveal disturbances in the structure of nephrons. On sections from control individuals, it was possible to distinguish nephron sections, namely: proximal, distal, and glomeruli, which were separated by islets of hematopoietic tissue (Figure 6a). In PAS staining, the proximal tubules had a distinct PAS-positive brush border.

Exposure to a concentration of 2 mg/L manifested in a significant enlargement of the sinusoidal spaces filled with erythrocytes, observed throughout the entire slide area, which may indicate hyperemia (Figure 6b). Additionally, an increase in the occurrence of neonephrogenic tubules was detected, the epitheliums of which had long oval nuclei showing few chromatins and a bright basophilic staining of the cytoplasm (Figure 6c). At this concentration, a slight decrease in the area of renal tubule epithelial nuclei was also observed (Figure 6i,j). At a concentration of 4 mg/L, in addition to the above changes, the presence of foci of inflammation located near the degradation sites of renal tubules was found (Figure 6d). Cubic epithelium with pycnotic nuclei were detected in these areas. The presence of narrowing of the lumen of some tubules, as well as an increase in the area of epithelial nuclei should be noted (Figure 6e).

Histopathological changes in the kidneys of *D. rerio* at 6 mg/L were comparable to those at lower concentrations. Changes in the structure of tubules were expressed in vacuolization of cubic epithelium, as well as an increase in the nuclei area (Figure 6f,g). In the hematopoietic tissue, foci of degradation were common and were expressed in the presence of pyknotic cells and also a small number of PAS-positive cells (Figure 6h).

**Figure 7:**
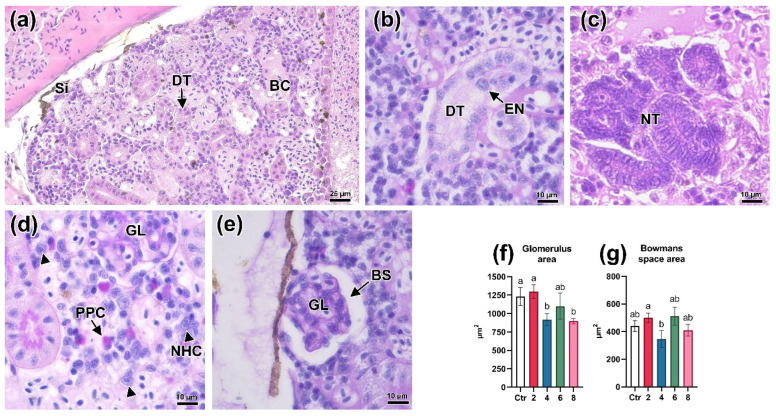
Effect of 8 mg/L BPA on nephron structure, hematopoietic tissue and morphometric parameters (**f**,**g**) of *Danio rerio* trunk kidney. (**a**–**e**): Significant circulatory disturbances manifested by sinusoidal dilation (Si) and blood congestion (BC); narrowing of the lumen and enlargement of the nuclei (EN) of the epithelium of some distal tubules (DT); increased occurrence of neonephrogenic tubules (NT); necrosis of areas of hematopoietic tissue associated with necrotic nuclei (NHC, arrowhead) and PAS-positive cells (PPC); decreased area of some glomeruli (GL) with an increase in Bowman’s space (BC). H&E (**a**,**c**) and PAS (**b**,**d**,**e**) staining. Scale bars of 25 μm (**a**) and 10 μm (**b**–**e**). Significance (*p* < 0.05) from the Kruskal–Wallis test. Superscript letters (a, b) indicate statistical significance between different experimental groups.

The histopathologic disturbances described for lower concentrations were most pronounced when exposed to 8 mg/L BPA. Circulation disorders, namely sinusoidal dilation and blood stasis were detected throughout the whole area of the organ (Figure 7a). Renal tubules showed signs of hypertrophy (Figure 7b), expressed by a significant increase in the area of the epithelial cell nucleus (*p* < 0.05; Figure 6i,j; Table S4). Thus, a large number of neonephrogenic tubules were found in the organ stroma (Figure 7c). Alterations of glomerulus morphology were manifested in a significant decrease in their area relative to the control at a concentration of 2 mg/L (*p* < 0.05; Figure 7f). At the same time, no differences in the area of Bowman’s space were revealed (Figure 7g).

Calculation of the histopathologic index showed that concentrations of 6 and 8 mg/L led to a significant increase in the number and severity of progressive (HIpc) and regressive (HIrc) changes (*p* < 0.05; Figure 6f; Table S5). These changes were predominantly expressed in alterations of hematopoietic tissue (presence of cells with pyknotic nuclei, increased number of PAS-positive cells, and general disturbances of organ histoarchitectonics) and structure of nephron sections (necrosis/inflammation of tubules, vacuolization of epithelium). Similar dynamics were also observed in the index of circulation disturbance (HIcd), which, at high concentrations of the toxicant, manifested itself in the expansion of sinusoidal spaces and blood congestion. It should be noted that the regressive changes at a concentration of 2 mg/L were comparable to the control and differed considerably from the value at higher concentrations.

### 3.6. Identifying the Concentration–Effect Relationship

**Figure 8:**
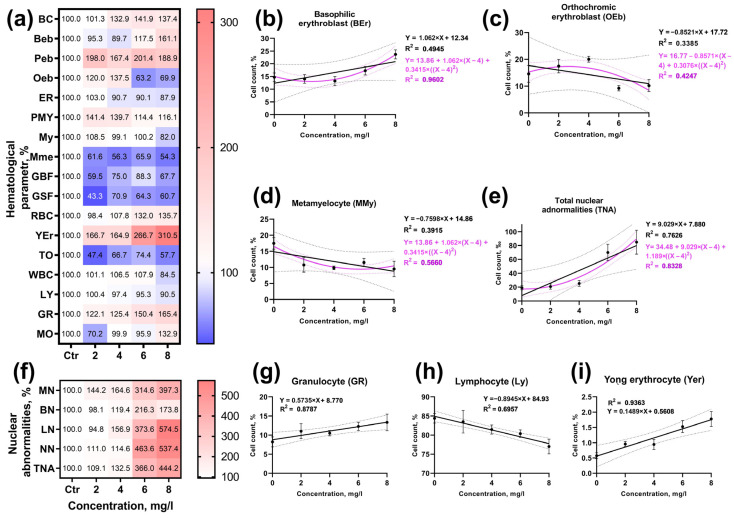
Concentration–effect relationship of BPA on hematological and hematopoietic parameters of *Danio rerio*. (**a**,**f**): matrix of changes in different parameters compared to the control in %; (**b**–**e**): comparison of linear and polynomial regression of hematopoietic parameters; (**g**–**i**): linear regression of hematological parameters.

To identify the concentration–effect relationship for the hematological and hematopoietic parameters of *D. rerio*, linear and polynomial regression analysis of the data with the most significant differences from the control was performed (Figure 5a,f). The most pronounced effect of BPA was on erythroid cell forms: the linear correlation coefficient for basophilic erythroblasts was 0.4945 (Figure 8b), and its value was considerably higher when a polynomial was used (R^2^ = 0.9602). The occurrence of orthochromatic erythroblasts was less dependent on BPA concentration in both linear and polynomial regression analyses (R^2^ = 0.3385 and 0.4247, respectively; Figure 8c). The number of metamyelocytes decreased as dependent on toxicant concentration, and this trend was more clearly demonstrated by the polynomial analysis (R^2^ = 0.5660; Figure 8d).

The occurrence rate of erythrocyte nuclear abnormalities (MN, LN, and NN) had a strict positive linear relationship with BPA concentration (Figure 8f; Figure S2). At the same time, the correlation between the total number of nuclear abnormalities (TNA) and toxicant concentration was more significant using polynomial analysis (R^2^ = 0.8328; Figure 8e). The parameters of the fish’s peripheral blood, such as the relative abundance of lymphocytes (R^2^ = 0.8787) and young erythrocytes (R^2^ = 0.9663), had a significant positive linear relationship with BPA concentration (Figure 8g,i). At the same time, the relative number of lymphocytes decreased with increasing toxicant concentration (R^2^ = 0.6357; Figure 8h).

**Figure 9:**
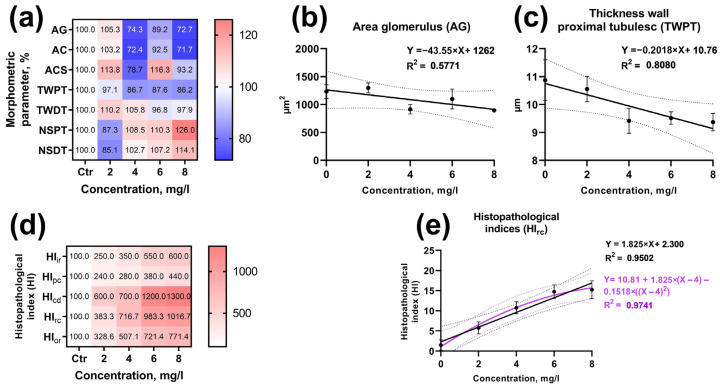
Concentration–effect relationship of BPA on histological parameters of *Danio rerio*. (**a**,**d**): matrix of changes in different parameters compared to the control in %; (**b**,**c**): linear regression; (**e**): comparison of linear and polynomial regression.

The linear regression model revealed a significant correlation between a number of *D. rerio* kidney and BPA concentration morphometric parameters (Figure 9a,d). Thus, the regression coefficient for the distal tubule wall thickness was 0.8080 (Figure 9c). In turn, the glomerular area had a smaller relationship with toxicant concentration (R^2^ = 0.5787; Figure 9b). A similar dependence was found for the other parameters. However, the application of the polynomial model did not demonstrate a more relevant regression coefficient.

The histopathological index of regressive changes, which had the largest contribution to the organ index, had comparable regression coefficients in both the linear (R^2^ = 0.9502) and polynomial regression analyses (R^2^ = 0.9741; Figure 9e). This is attributed to the similar frequency and prevalence of histopathologic abnormalities at BPA concentrations of 6 and 8 mg/L.

For other studied parameters, the application of linear and polynomial regression analysis did not reveal a significant relationship with the studied BPA concentrations.

## 4. Discussion

### 4.1. Acute BPA Toxicity to Adult Zebrafish

Obtained data made it possible to determine the acute toxic effect limits of BPA for adult zebrafish. Concentrations exceeding 4 mg/L reliably resulted in fish mortality within 96 h, with an LC50 of 6.22 mg/L. Acute toxicity of Bisphenol A was observed with fish mortality during the first 48 h of the experiment, with no further mortality occurring between 48 and 96 h. The lethal concentration in this study aligns with data from other researchers. For instance, medaka (*Oryzias latipes*) exhibited an LC50 of 9.4 mg/L for 96 h [48], while tilapia (*Oreochromis mossambicus*) showed an LC50 of 6.48 mg/L [49]. Carp (*Cyprinus carpio*) had an LC50 of 4.6 mg/L [50], and the spotted snakehead (*Channa punctatus*) had an LC50 of 7.62 mg/L [51].

In the case of the early developmental stages of zebrafish (embryo and larva), half of the individuals died within 72 h at concentrations of 8.22 and 10.94 mg/L, respectively [28]. These values are slightly higher than the ones obtained in this study for adult fish. The variations in lethal concentration values may be attributed to differences in fish species characteristics and the use of different solvents, such as acetone.

Based on the results of the histological study, the cause of fish mortality was attributed to the systematic toxic effects of BPA on hematopoiesis and excretory functions. In addition to kidney disorders, visible signs of gill lamellae atrophy were observed (see Figure S3). Similar mechanisms of action have been reported for many toxicants, including heavy metals, pesticides, and nanoparticles, which exhibit acute toxicity at high concentrations [52]. In surviving individuals, as a result of the adaptation processes, changes in the composition of the hematopoietic tissue cells and peripheral blood were observed.

### 4.2. Hematopoietic Parameters of the Head Kidney

The morphological stages of erythroid cell development found in the head kidney of zebrafish align with the descriptions provided by earlier authors [37,53,54]. However, it is worth noting that some morphological differences in myeloid cells, specifically in promyelocytes and myelocytes, were comparable to the descriptions by Kondera et al. [37]. In this study, myelocytes exhibited the largest size among all granulocyte precursors, which deviates from Kondera’s description, but consistent with the one reported by Ivanova [35].

The relative abundance of early stages of erythrocyte differentiation observed in smears of zebrafish head kidney is consistent with findings from previous studies. For instance, the numbers of polychromatic and orthochromatic erythroblasts obtained in this study (13.58% and 14.58%) are in agreement with data from several researchers [37,38,54,55]. These studies reported similar ranges, with percentages varying from 12.78% to 19.36% for polychromatic erythroblasts and 7.12% to 12.84% for orthochromatic erythroblasts. Notably, a significant variation was observed in the occurrence of mature erythrocytes (ranging from 0.36% to 12.44%), which is likely attributed to differences in the methodology used for preparing head kidney smears. The occurrence of myeloid hematopoietic progenitor cells also aligns with previous data [56,57,58], demonstrating an incremental increase as cells progress through the differentiation stages.

It is also important to consider that the variations in the frequency of hematopoietic progenitor cells occurrence may be attributed to species-specific differences [13,15,37] as the mentioned studies encompass a wide range of species (such as *Cyprinus carpio*, *Ictalurus punctatus*, *Acipenser dabryanus*, *Scorpaena porcus*, etc.).

Despite significant advancements in the study of hematopoiesis in lower vertebrates, including fish, there is an ongoing need for further refinement of morphological and quantitative assessment methods [17]. In our study, we introduced an approach for counting hematopoietic progenitor cells, with particular emphasis on elements from the erythropoietic and myelopoietic series. However, it is important to note that our myeloid cell counting did not involve categorization based on staining affinity (neutrophils, basophils, eosinophils). This described approach has the potential for application in evaluating the effects of toxic substances on hematopoiesis.

The conducted study allows us to conclude that BPA, at concentrations of 6 and 8 mg/L, influences the differentiation of cell elements in the erythroid series. Moreover, the impact of low concentrations of the toxicant was observed on various cell types, including orthochromatic erythroblasts, metamyelocytes, and differentiated forms of granulocytes. The increase in erythropoiesis activity as a response to stress factors, including toxic exposure, is a common phenomenon observed in all vertebrate organisms. An extensively studied factor affecting the number of red blood cells in peripheral blood is anemia, which triggers the release of stored red blood cells and an increased proliferation of new ones, consequently enhancing blood oxygen saturation [14,59]. Given that the primary route of BPA entry into a fish’s body is through the gills [6], it can be hypothesized that the disruption of their function due to toxic effects might lead to anemia and, in turn, stimulate erythropoiesis.

Oxidative stress could represent another mechanism of BPA’s toxic effects on erythropoiesis. Previous studies have demonstrated that BPA-induced inflammation directly correlates with an increase in the levels of reactive nitrogen and oxygen species [12,60]. The excess of these free radicals has the potential to cause cytotoxic damage to various body tissues, including blood cells and the hematopoietic tissue of zebrafish. This phenomenon may also account for the reduced occurrence of certain myeloid cells across all experimental BPA concentrations, as they may migrate to sites of nonspecific immune response. Given that granulocytes in fish have a short life cycle and a high renewal rate [58], prolonged exposure to BPA may result in significant alterations in their occurrence within the hematopoietic tissue of the head kidney.

The endocrine-disruptive effects of BPA, particularly its estrogenic activity [9], may also extend to the impairment of the immune-endocrine components through its impact on the hypothalamus–pituitary–adrenal axis, which plays a role in the regulation of myeloid and lymphoid cell activity [61,62]. In fish, the head kidney serves multiple functions, including hematopoietic, excretory, and potentially endocrine functions (e.g., the adrenal gland [63]). The disruption of these functions, along with other elements of the head kidney, could amplify the toxic effects of BPA.

Similar alterations in erythropoiesis, resembling signs of anemia resulting from cytotoxic damage to red blood cells, have been observed with other toxicants such as cadmium [64], copper nanoparticles [65], dioxin [66], and nitrite [14].

### 4.3. Genotoxicity

Numerous studies have demonstrated the genotoxic effects of BPA on both aquatic and higher vertebrates [7,51,67,68]. In this study, using a micronucleus test on fish erythrocytes, acute exposure to BPA at concentrations of 6 and 8 mg/L resulted in a significant increase in the frequency of nuclear abnormalities. Examination of nuclear abnormalities revealed that lobed nuclei and notched nuclei were significantly more common than other types of alterations. The absence of significant differences from the control at concentrations of 2 and 4 mg/L suggests the lack of a genotoxic effect in the conditions of short-term experiments. However, in long-term experiments, these concentrations could also lead to disruptions in the nuclear apparatus of erythrocytes.

For instance, Akram et al. [8] demonstrated that the number of nuclear abnormalities in a BPA concentration of 1.5 mg/L significantly increased only after 45 to 60 days of exposure. Similarly, in the study by Afzal et al. [7], a meaningful increase in the number of erythrocytes with nuclear abnormalities was observed at concentrations greater than 3 mg/L after 30 days of exposure. In an acute experiment lasting 96 h, BPA resulted in a significant increase in micronuclei frequency in *Channa punctatus* at concentrations of 1.91 and 3.81 mg/L [51]. The researchers mentioned above attribute the genotoxicity of BPA to increased free radical generation and oxidative stress. The variations in the genotoxic effect of BPA may be related to species-specific differences in erythropoiesis and the sensitivity of fish.

The formation of nuclear abnormalities occurs as a result of cell division from acentric chromosome fragments and/or chromatid fragments in the anaphase of mitosis [25], and the frequency of these abnormalities may be regulated by the rate of erythropoiesis [23]. Thus, the increase in blast forms of erythrocytes in the head kidney may be directly related to the increased frequency of abnormal erythrocytes in the peripheral blood of zebrafish. According to some authors [69,70], the formation of nuclear abnormalities after exposure to a toxicant typically occurs within 1–2 days. This suggests that visible abnormalities of the nuclear apparatus occur at the latter stages of erythrocyte differentiation. In our study, basophilic and polychromatic erythroblasts did not exhibit morphologic disturbances in the nucleus, even when exposed to concentrations of 6 and 8 mg/L. This might be due to the fact that the MN can be reincorporated into the main nucleus during subsequent mitosis or destroyed by the autophagy process [71,72].

The increased frequency of nuclear anomalies, including lobed nuclei and notched nuclei, detected in the study may indicate the effect of BPA on the frequency of structural chromosomal aberrations (i.e., clastogenesis). One of the main mechanisms of action of clastogenic agents is the breaking of DNA strands, which, in the case of incorrect repair, are able to form acentric chromatids [73]. Toxicants that induce oxidative stress with short-term action have been shown to specifically lead to clastogenic abnormalities [74]. The ability to induce nuclear disorders has also been observed for other compounds in the phenolic group [75].

The results obtained in this study demonstrate that considering several morphological types of nuclear abnormalities in the blood of zebrafish allows for the simultaneous screening of pollutants in conjunction with other hematological indicators.

### 4.4. Peripheral Blood Composition

Under stress or toxic effects, changes in the composition of peripheral blood occur most rapidly. The compensatory response of the fish organism to the negative effects of stress factors is manifested in the release of cellular elements, hormones, and enzymes into the peripheral blood and changes in its chemical composition [76]. In our study, exposure to high concentrations of BPA resulted in significant changes in both the number of red blood cells and the leukocyte ratio. Thus, the observed fluctuations in the number of RBCs and the relative occurrence of immature erythrocytes directly correlate with data on changes in the relative occurrence of erythroblasts in the head kidney of zebrafish.

The release of immature forms of erythrocytes only partially compensated for the toxic effects of high BPA concentrations, leading to systemic disorders and the death of some fish by the end of the acute experiment. In contrast, at concentrations of 2 and 4 mg/L, no such effect was observed, suggesting that the fish organism can withstand such pollutant levels during short-term exposure. Existing studies present conflicting data regarding the effect of BPA on the number of red and white blood cells. For example, in *Oncorhynchus mykiss* there is an increase in RBC counts, while in *Vimba vimba* [77] and *Channa punctatus* [51] there is a decrease. It is possible that the toxicity of BPA increases with rising temperatures in the aquatic environment.

Changes in the composition of white blood elements, while accompanied by an increase in the number of granulocytes and monocytes at high concentrations, did not lead to a significant increase in the WBC count in the peripheral blood of fish. On the contrary, their number significantly decreased at a concentration of 8 mg/L. This decrease may be related to the disruption of differentiation processes in hematopoietic organs and/or their mobilization to participate in nonspecific immune responses in other tissues. Similar results were obtained by Holeyappa et al. [78] in *Cyprinus carpio* under chronic exposure to BPA.

The number of thrombocytes significantly decreased at concentrations of 2, 4, and 8 mg/L. The decrease in their quantity at the lowest concentrations may have been caused by disruptions in the blood coagulation system. However, it is important to note that, as pointed out by Witeska [79], blood coagulation under stress is not always directly related to an increase in the number of thrombocytes in the bloodstream of fish.

### 4.5. Histological Parameters of Head and Trunk Kidney

Examination of the histological structure of the head kidney of zebrafish revealed the presence of pathological abnormalities at all studied concentrations. Significant disturbances in the structure of hematopoietic tissue, which were observed starting from the concentration of 4 mg/L, are directly associated with changes in the ratio of hematopoietic cells. The most notable pathological changes included impaired circulation and the occurrence of inflammation/necrosis foci. Nephrotoxic effects of BPA have also been reported by many authors across a wide range of concentrations. Among the histopathologic abnormalities of fish hematopoietic tissue, necrosis and a reduction in the amount of hematopoietic tissue have been identified [7,78,80] which is in agreement with our results. As noted earlier, the action of BPA on hematopoietic tissue is primarily associated with the induction of oxidative stress. The excretion of BPA as part of bile acids through the intestine [81] and the low toxicity of its metabolites [82] suggest that renal damage is associated with the persistence of its activity in blood and tissue fluids. Additionally, renal impairment may be related to the continuous ingestion of the toxicant through the gills from the surrounding aquatic environment [6].

The kidney plays a crucial role in excretion and osmoregulation and has been reported by several researchers to be primarily affected by toxicants from the phenolic group [83]. Toxicants enter the kidney through the bloodstream [84]. In addition to its impact on hematopoietic tissue, BPA also affected the structure of nephrons. Even at a concentration of 2 mg/L, significant circulatory alterations were observed, which could be attributed to hyperemia. As the concentration of the toxicant increased, circulatory alterations progressed, potentially indicating disturbances in the integrity of vascular and capillary walls. Changes in renal tubules, such as the enlargement of cubic epithelial nuclei, vacuolization, and the narrowing of the tubule lumen, represent the initial response of the tissue to the presence of a toxicant in the filtrate [85]. Similar histopathologic changes have been observed during exposure to various pollutants.

The increased occurrence of neonephrogenic tubules under the influence of BPA suggests the activation of renal tissue regeneration mechanisms. According to some authors [86], these areas are where cell proliferation occurs to form new renal tubules. However, the supposed induction of neonephrogenesis under the influence of BPA warrants further study.

The head portion of the trunk kidney, which combines hematopoietic and excretory functions, is particularly susceptible to the acute effects of BPA. Therefore, it can be concluded that high concentrations of BPA have an acute nephrotoxic effect on zebrafish.

### 4.6. Concentration–Effect Relationship

Polynomial regression analysis can, in certain cases, unveil more intricate relationships between the concentration of a toxicant and changes in a test parameter than linear regression [87]. Polynomial regression is actively employed in determining the extent of heavy metal accumulation in the tissues of aquatic organisms [88], as well as in establishing the most effective concentrations of feed additives [89].

Utilizing this method, it was observed that, for some immature forms of erythrocytes, a correlation existed between BPA concentration and the relative cell number. This correlation was marked by the absence of BPA influence at low/sublethal concentrations (2 and 4 mg/L) and a significant increase or decrease in the number of cells at higher/lethal concentrations (6 and 8 mg/L). This pattern was particularly pronounced for basophilic erythroblasts and the total number of nuclear abnormalities, both of which showed a significant increased at higher concentrations of the toxicant.

In the case of parameters such as the number of nuclear abnormalities and the relative abundance of lymphocytes, granulocytes, and young erythrocytes, linear regression analysis revealed a significant concentration–effect relationship. Additionally, a linear dependence on the concentration of BPA was found for certain morphometric kidney parameters. It is worth noting that the presence of a linear relationship between the occurrence of nuclear abnormalities and the concentration of the toxicant suggests the presence of clastogenic genotoxicity of BPA [90].

The obtained results indicate that the acute toxic effect of BPA is most pronounced at high concentrations (6 and 8 mg/L). However, for several parameters, such as the number of young erythrocytes and the relative number of lymphocytes, a linear dependence on the concentration of the toxicant was observed. Similar linear concentration-dependent effects were also noted when studying the impact of BPA on hematological parameters in *Oncorhynchus mykiss* and *Vimba vimba* [77].

According to Canesi et al. [6], a nonlinear dose–response relationship is often observed for compounds with endocrine-disruptive effects. Additionally, toxicogenomics data indicate that BPA is characterized by an inverted U-shaped pattern in the dose–response relationship [6], which is partially supported by the results obtained in this work. To establish more accurate relationships between BPA concentration and the indicators of aquatic organisms, further research is needed, encompassing both low concentrations (sublethal/occurring in the environment) and high concentrations.

Differences in the effects of BPA concentrations on the composition of hematopoietic cells may be attributed to variations in the timing and stages of differentiation of blast forms in the erythroid and myeloid lineages. This variability can also be explained by the inhibitory effects of low concentrations of BPA and the cytotoxic effects of higher concentrations. However, the differentiation of individual hematopoietic tissues occurs at different rates, and as a result, short-term exposure to a stress factor could lead to a significant shift in their ratio [91].

### 4.7. Hematological Parameters in the Evaluation of Endocrine-Disruptive Substances

The results obtained in this work and the data from other researchers show that BPA can have a significant effect on the processes of hematopoiesis. Exposure to high concentrations of BPA disturbs the processes of development and function of fish blood cells, which is expressed not only in changes in the kinetics of blood development and formation of nuclear anomalies in erythrocytes but also in the appearance of histopathological disorders in hematopoietic organs (head kidney). Thus, these abnormalities may reduce the ability of fish to respond to infections and other physiological stressors [14,54]. In addition, due to the fact that BPA is able to accumulate in tissues, it poses a danger not only to aquatic animals but also to humans (through diet) [4,92].

For these reasons, when searching for new ways to alleviate the toxic effect of endocrine-disruptive substances (e.g., the use of antioxidants, sorbents, probiotics, food/feed additives) [93,94,95], the indicators of the cellular composition of hematopoietic organs and peripheral blood can act as significant test parameters.

## 5. Conclusions

Bisphenol A at concentrations of 6 and 8 mg/L resulted in the mortality of a portion of *Danio rerio* individuals during the acute experiment (96 h). This mortality can be attributed to systemic toxic effects, which include disruptions in hematopoiesis and osmoregulation. These concentrations also led to a substantial increase in the occurrence of erythrocyte nuclear abnormalities, indicating a potential genotoxic effect. Notably, significant changes in the composition of hematopoietic cells were observed in head kidney smears across all studied BPA concentrations. These changes are in line with alterations in the number of cells in peripheral blood and the histopathologic changes observed in the hematopoietic tissue of zebrafish head kidney. This alignment is further confirmed through the calculation of histopathologic indices.

The identified toxic effects of BPA, as described using both linear and polynomial models, revealed relatively weak responses to low concentrations of the toxicant, with a sharp change occurring at concentrations of 6 and 8 mg/L. This suggests that the limits of adaptation responses in zebrafish are within the range of 4–6 mg/L, slightly below the obtained LC50 (96 h) values. In summary, the results of this study demonstrate that the systemic effects of high BPA concentrations primarily manifest in hematopoietic organs and the cell composition of the blood.

## Figures and Tables

**Table 1:** Mean mortality of *Danio rerio* (in %) in the acute experiment during exposure in BPA solutions and LC50 values.

Concentration, mg/L	Survival Rate, %				LC50 Value (CI), mg/L
	12 h	24 h	48 h	96 h	
Control	100	100	100	100	LC_50_^12^ = 18.04 (17.81–18.28); LC_50_^24^ = 7.55 (7.38–7.72); LC_50_^48/96^ = 6.22 (6.11–6.33)
0.5	100	100	100	100	
1	100	100	100	100	
2	100	100	100	100	
4	100	100	96.6	96.6	
6	100	83.3	56.6	56.6	
8	100	40	13.3	13.3	
12	100	0	0	0	
16	76.6	0	0	0	
20	26.6	0	0	0	

**Table 2:** Relative abundance of cellular elements in smears of hematopoietic tissue of *Danio rerio* head kidney during acute exposure to Bisphenol A. Significance (*p* < 0.05) from the Kruskal–Wallis test. Superscript letters (a–c) indicate statistical significance between different experimental groups.

Hematopoetic Cell, %	BPA Concentration, mg/L				
	Ctr	2	4	6	8
Blast cells (BC)	1.55 ± 0.15 ^b^	1.57 ± 0.26 ^ab^	2.06 ± 0.86 ^ab^	2.2 ± 0.39 ^a^	2.13 ± 0.32 ^a^
Basophilic erythroblast (BEb)	14.7 ± 1.66 ^b^	14.02 ± 1.7 ^b^	13.19 ± 1.8 ^b^	17.29 ± 1.7 ^ab^	23.7 ± 1.79 ^a^
Polychromatic erythroblast (PEb)	13.58 ± 1.7 ^b^	26.9 ± 2.88 ^a^	22.7 ± 1.07 ^ab^	27.35 ± 0.92 ^a^	25.65 ± 3.8 ^a^
Orthochromatic erythroblast (OEb)	14.58 ± 3.1 ^ab^	17.49 ± 2.5 ^a^	20.04 ± 1.09 ^a^	9.22 ± 1.01 ^b^	10.19 ± 2.2 ^b^
Erythrocyte (ER)	7.34 ± 1.45	7.56 ± 0.55	6.66 ± 0.81	6.61 ± 0.91	6.45 ± 1.13
Promyelocyte (PMy)	1.74 ± 0.39	2.46 ± 0.96	2.43 ± 0.71	1.99 ± 0.08	2.02 ± 0.45
Myelocyte (My)	6.37 ± 1.11	6.91 ± 1.63	6.31 ± 0.69	6.38 ± 1.04	5.22 ± 0.68
Metamyelocyte (MMy)	17.4 ± 1.78 ^a^	10.76 ± 2.2 ^b^	9.83 ± 0.58 ^b^	11.5 ± 1.28 ^ab^	9.49 ± 2.4 ^b^
Granulocyte-band form (GBF)	8.36 ± 1.48 ^a^	4.97 ± 0.9 ^c^	6.2 ± 0.42 ^abc^	7.38 ± 1.26 ^ab^	5.66 ± 0.63 ^c^
Granulocyte-segmented form (GSF)	11 ± 1.38 ^a^	4.78 ± 0.97 ^c^	7.83 ± 0.65 ^ab^	7.11 ± 0.71 ^bc^	6.71 ± 0.94 ^bc^
Unclassified cells	3.14 ± 0.18	2.5 ± 0.5	2.54 ± 1.17	2.87 ± 0.79	2.68 ± 0.21

## Data Availability

Data are contained within the article and Supplementary Materials.

## Supplementary Materials

The following supporting information can be downloaded at: https://www.mdpi.com/article/10.3390/ani13233685/s1, Table S1: Cell and nucleus size of various hematopoietic cells in control *Danio rerio*; Figure S1: Relative abundance of cellular elements of hematopoietic tissue of *Danio rerio* head kidney during acute exposure to Bisphenol A; Table S2: Number and relative occurrence of cellular elements of peripheral blood of *Danio rerio* during acute exposure to Bisphenol A; Table S3: Relative abundance of *Danio rerio* erythrocytes with nuclear abnormalities under acute exposure to Bisphenol A; Table S4: Morphometric parameters of kidney of experimental *Danio rerio* under acute exposure to bisphenol A; Table S5: Histopathological indices of kidney of experimental *Danio rerio* under acute exposure to bisphenol A; Table S6: Abbreviation of histopathological indexes and morphometric parameters used in the work; Figure S2: Linear concentration–effect relationship of BPA on nuclear abnormalities of *Danio rerio*; Figure S3: Histological sections of *Danio rerio* gills in control and experimental groups. (a) control group gills, including primary (PL) and secondary lamellae (SL), as well as basal cells (BC) and respiratory epithelium; (b) 6 mg/L: at this concentration, a decrease in the thickness of secondary lamellae and respiratory epithelium (RE) as well as basal cells was observed; (c, d) 8 mg/L: in addition to the above-mentioned abnormalities, areas with curvature of secondary lamellae (LC) were observed. H&E staining Scale bars 25 μm.
