# Supplementary material for: The Effects of Acute Bisphenol A Toxicity on the Hematological Parameters, Hematopoiesis, and Kidney Histology of Zebrafish (Danio rerio)
Source: Animals (Basel). 2023 Nov 28;13(23):3685. doi: 10.3390/ani13233685 (PMC10705224; doi:10.3390/ani13233685)
Supplement: Supplementary file 1 [file animals-13-03685-s001.zip › animals-2720777-SI.pdf]

Table S1. Cell and nucleus size of various hematopoietic cells in control *Danio rerio*.

Cell type	Nuclei		Cell	
	length	width	length	width
Blast cells (BC)	8.04±0.5	7.23±1.09	10.5±0.97	10.53±0.63
Basophilic erythroblast (BEb)	5.73±0.54	5.8±0.51	8.46±0.76	8.22±0.88
Polychromatic erythroblast (PEb)	5.13±0.7	4.35±0.91	7.75±0.84	6.72±1.07
Orthochromatic erythroblast (OEb)	3.01±0.5	4.3±0.55	7.26±1.09	5.66±0.97
Erythrocyte (ER)	1.83±0.22	3.96±0.48	7.3±0.78	4.61±0.6
Promyelocyte (PMy)	4.65±0.56	6.67±1.04	8.53±0.8	7.53±0.84
Myelocyte (My)	6.9±0.93	6.75±0.93	10.16±0.88	9.8±0.93
Metamyelocyte (MMy)	3.29±0.61	5.35±0.73	7.91±0.58	7.01±0.82
Granulocyte-band form (GBF)	7.95±1.1	5.43±1.3	10.05±0.55	10.46±0.95
Granulocyte-segmented form (GSF)	7.87±1.15	9.68±1.24	10.36±0.71	11.15±1.41

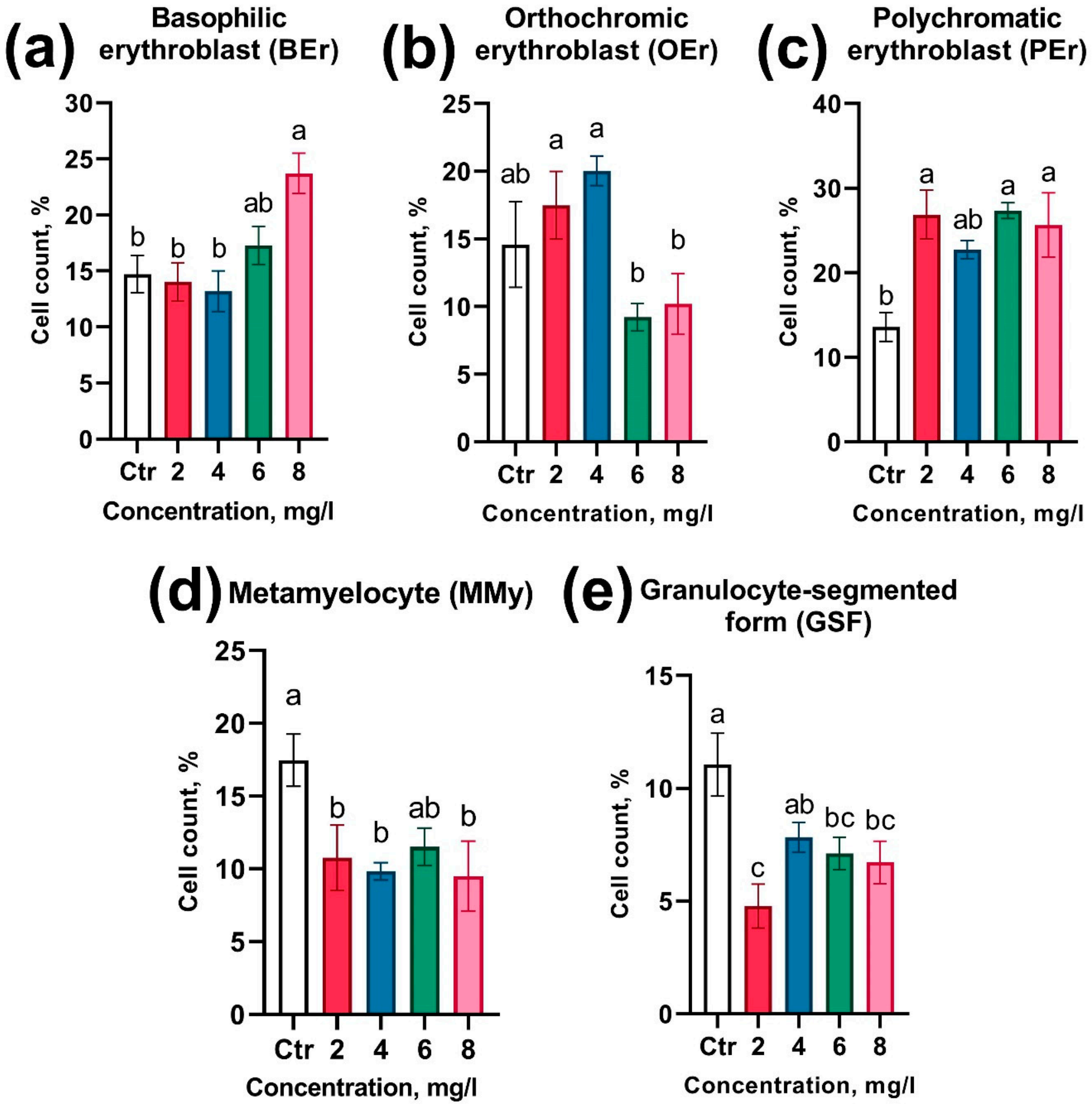

Figure S1. Relative abundance of cellular elements of hematopoietic tissue of *Danio rerio* head kidney during acute exposure to Bisphenol A. Significance ($p < 0.05$) from the Kruskal-Wallis test. Superscript letters (a-c) indicate statistical significance between different experimental groups.

Table S2. Number and relative occurrence of cellular elements of peripheral blood of *Danio rerio* during acute exposure to Bisphenol A. Significance ($p < 0.05$) from the Kruskal-Wallis test. Superscript letters (a-c) indicate statistical significance between different experimental groups.

Parametr	BPA concentration, mg/L				
	Ctr	2	4	6	8
RBC, 10^6/µL	3.19 ± 0.28^{b}	3.14 ± 0.26^{b}	3.44 ± 0.43^{ab}	4.21 ± 0.41^{a}	4.33 ± 0.62^{a}
Yong erythrocyte (YER), %	0.57 ± 0.09^{c}	0.95 ± 0.08^{bc}	0.94 ± 0.16^{bc}	1.52 ± 0.19^{ab}	1.77 ± 0.24^{a}
Trombocyte (TO), %	0.78 ± 0.05^{a}	0.37 ± 0.07^{c}	0.52 ± 0.12^{bc}	0.58 ± 0.05^{ab}	0.45 ± 0.06^{bc}
WBC, 10^4/µL	$7.56\pm0.28a^{b}$	7.64 ± 0.42^{ab}	8.05 ± 0.4^{a}	8.16 ± 0.64^{a}	6.39 ± 0.62^{b}
Lymphocyte (LY), %	84.12 ± 1.37^{b}	84.44 ± 2.78^{b}	80.78 ± 4.6^{ab}	81.28 ± 1.3^{ab}	76.13 ± 1^{b}
Granulocyte (GR), %	8.49 ± 1.43^{b}	10.37 ± 1.85^{ab}	10.48 ± 0.56^{ab}	12.95 ± 0.42^{a}	14.04 ± 1.98^{a}
Monocyte (MO), %	7.38 ± 0.85^{ab}	5.18 ± 0.92^{b}	8.73 ± 4.54^{ab}	5.75 ± 1.23^{ab}	9.81 ± 2.04^{a}

Table S3. Relative abundance of *Danio rerio* erythrocytes with nuclear abnormalities under acute exposure to Bisphenol A. Significance ($p < 0.05$) from the Kruskal-Wallis test. Superscript letters (a-c) indicate statistical significance between different experimental groups.

Nuclear adnormalities, ‰	BPA concentration, mg/L				
	Ctr	2	4	6	8
Micronuclei (MN)	2.6 ± 1.7^{c}	3.7 ± 1.8^{c}	4.2 ± 0.9^{bc}	8.1 ± 1.5^{ab}	10.3 ± 2.6^{a}
Blebed nuclei (BN)	4.2 ± 3.1^{ab}	4.1 ± 1.2^{b}	5.1 ± 0.9^{ab}	9.1 ± 2.2^{a}	7.3 ± 1.6^{ab}
Lobbed nuclei (LN)	4.4 ± 1.9^{b}	4.2 ± 1.3^{b}	6.9 ± 2.5^{b}	16.5 ± 3.4^{a}	25.4 ± 5.9^{a}
Notched nuclei (NN)	7.7 ± 0.5^{b}	8.6 ± 2.3^{b}	8.8 ± 2.4^{b}	35.9 ± 6.5^{a}	41.6 ± 8.3^{a}
Total nuclear adnormalities (TNA)	19.1 ± 3^{b}	20.8 ± 3.8^{b}	25.3 ± 4.2^{ab}	69.9 ± 11.8^{a}	84.8 ± 17.2^{a}

Table S4. Morphometric parameters of kidney of experimental *Danio rerio* under acute exposure to bisphenol A.

Morhometric parametr	Experemental group				
	CTR	2	4	6	8
Area glomerulus	1232.21±123.6	1297.03±90.45	915.15±83.43	1099±178.13	895.94±35.73
Area corpuscle	1696.83±114.39	1750.52±94.26	1227.59±95.56	1569.57±215.91	1215.88±123.03
Area Bowmans space	440.24±39.14	501.01±34.04	346.55±61.56	512.06±65.28	410.38±41.69
Thickness wall proximal tubules	10.87±0.73	10.55±0.45	9.42±0.45	9.52±0.22	9.37±0.31
Thickness wall distal tubules	9.2±0.31	10.14±1.3	9.74±0.86	8.9±0.58	9.01±0.41
Nuclei squre proximal tubulesc	12.42±0.62	10.85±0.49	13.47±0.67	13.7±0.82	15.64±0.43
Nuclei squre distal tubules	12.94±0.77	11.01±0.24	13.29±1.56	13.87±1.73	14.76±0.97

Table S5. Histopathological indices of kidney of experimental *Danio rerio* under acute exposure to bisphenol A.

Alteration	CTR	2	4	6	8
Inflammatory responses	0.5±0.58	1.25±0.5	1.75±0.5	2.75±0.96	3±0.82
Progressive changes	1.25±1.5	3±0.82	3.5±1.29	4.75±0.96	5.5±1.29
Circulatory disorders	0.25±0.5	1.5±0.58	1.75±0.5	3±0.82	3.25±0.5
Regressive changes	1.5±1.29	5.75±1.5	10.75±1.5	14.75±1.71	15.25±2.22
Organ index	3.5±1.29	11.5±1.73	17.75±0.96	25.25±1.5	27±2.58

Table S6. Abbreviation of histopathological indexes and morphometric parameters used in the work.

Abbreviation	Deciphering
AB	Area Bowmans Space
AC	Area Corpuscle
AG	Area Glomerulus
BC	Blast Cells
BC	Blood Congestion
Beb	Basophilic Erythroblast
BN	Blebbed Nuclei
BPA	Bisphenol A
BV	Blood Vessels
CI	Confidence Intervals
DMSO	Dimethyl Sulfoxide
DT	Distal Tubules
EN	Enlarged Nuclei
ER	Erythrocyte
GBF	Granulocyte-Band Form
GL	Glomeruli
GR	Granulocyte
GSF	Granulocyte-Segmented Form
H&E	Hematoxylin And Eosin
HI	Histopathologic Index
HIcdI	Circulatory Disorders
HIioI	Organ Index
HIirI	Histopathological Index Inflammation Response
HIpcI	Progressive Changes
HIrcI	Regressive Changes
HT	Hematopoietic Tissue
If	Inflammation
LC50	Lethal Concentration
LN	Lobbed Nuclei
LY	Lymphocyte
Ly	Lymphocyte-Like Cells
MN	Micronuclei
MO	Monocyte
My	Myelocyte
Ne	Necrosis
NHC	Necrotic Cells
NN	Notched Nuclei
NSDT	Nuclei Squre Distal Tubules
NSPT	Nuclei Squre Proximal Tubulesc
Oeb	Orthochromatic Erythroblast
PAS	Periodic Acid-Schiff
Peb	Polychromatic Erythroblast
Pmy	Promyelocyte

PN	Pycnotic Nuclei
PPC	PAS-Positive Cells
PT	Proximal Tubules
RBC	Red Blood Cells
RC	Renal Capsule
Si	Sinusoidal Spaces
TNA	Total Nuclear Adnormalities
TO	Trombocyte
TWDT	Thickness Wall Distal Tubules
TWPT	Thickness Wall Proximal Tubules
UN	Unclassified Cells
VA	Vacuolization Of Renal Tubule Epithelium
WBC	White Blood Cells
YER	Yong Erythrocyte
Mмy	Metamyelocyte

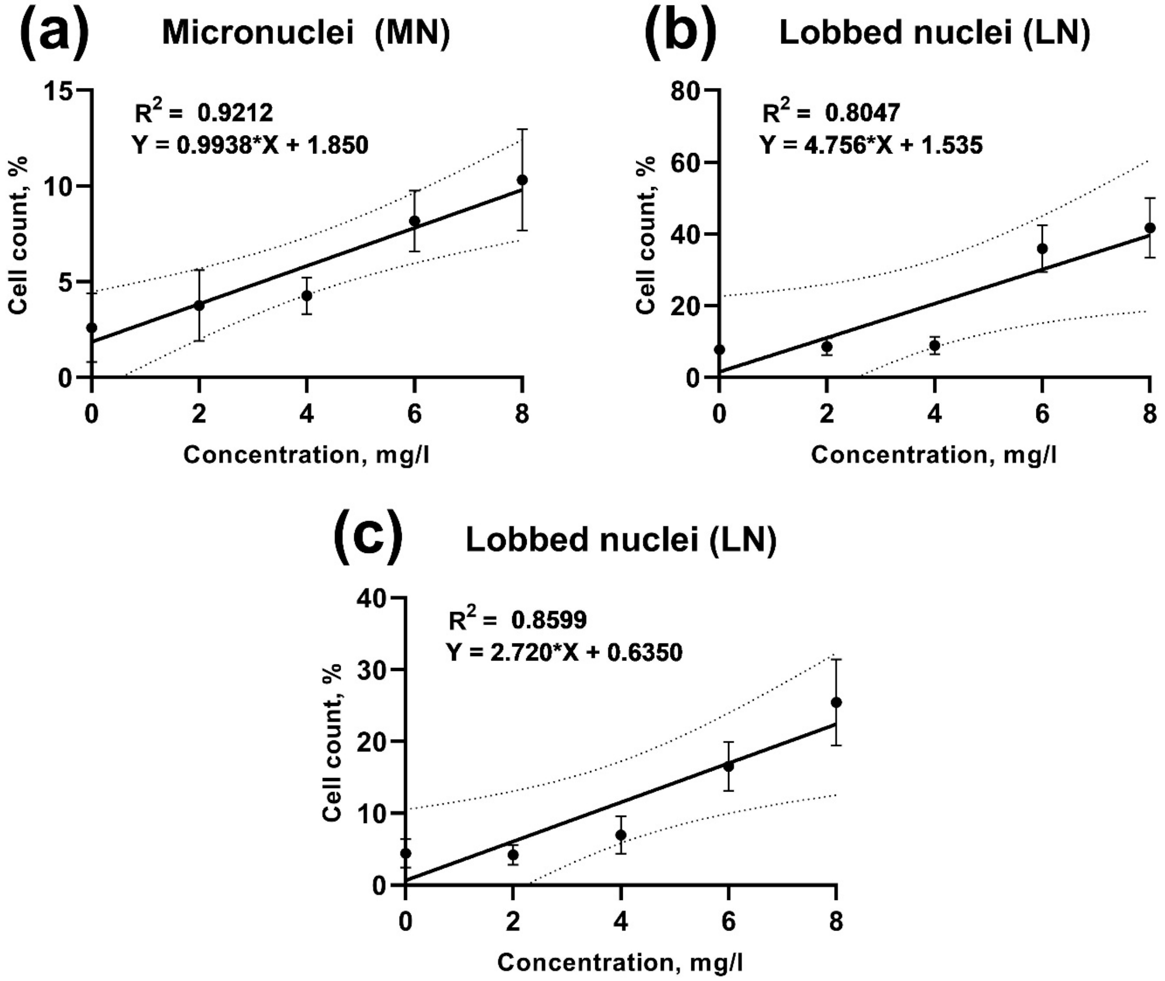

Figure S2. Linear concentration-effect relationship of BPA on nuclear abnormalities of *Danio rerio*

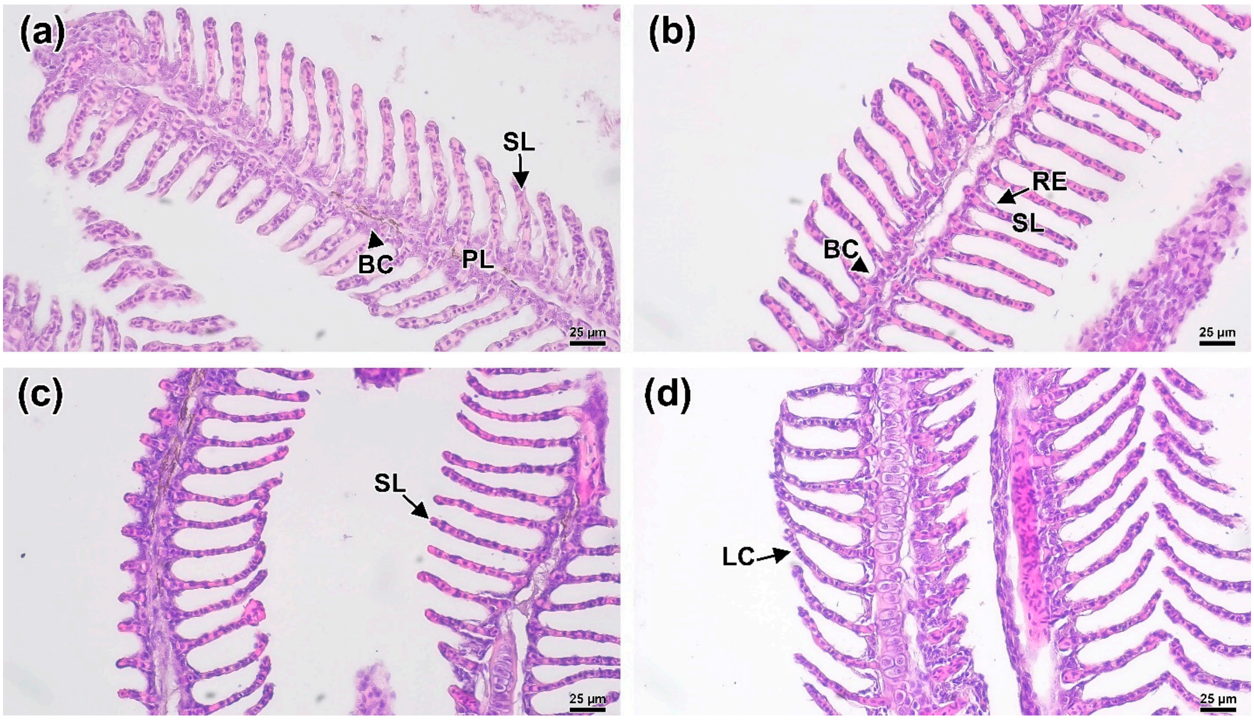

Figure S3. Histological sections of *Danio rerio* gills in control and experimental groups. (**a**) control group gills, including primary (PL) and secondary lamellae (SL), as well as basal cells (BC) and respiratory epithelium; (**b**) 6 mg/L: at this concentration, a decrease in the thickness of secondary lamellae and respiratory epithelium (RE) as well as basal cells was observed; (**c, d**) 8 mg/L: in addition to the above-mentioned abnormalities, areas with curvature of secondary lamellae (LC) were observed. H&E staining Scale bars 25 µm.
